# Diffusion-weighted imaging of suspicious (BI-RADS 4) breast lesions: stratification based on histopathology

**DOI:** 10.1590/0100-3984.2015.0224

**Published:** 2017

**Authors:** João Ricardo Maltez de Almeida, André Boechat Gomes, Thomas Pitangueira Barros, Paulo Eduardo Fahel, Mario de Souza Rocha

**Affiliations:** 1 MD, Radiologist, Department of Diagnostic Imaging, Clínica de Assistência à Mulher - Grupo CAM, Salvador, BA, Brazil.; 2 BMSc, Clínica de Assistência à Mulher - Grupo CAM, Department of Biomedicine, Escola Bahiana de Medicina e Saúde Pública – Campus Brotas, Salvador, BA, Brazil.; 3 MD, Pathologist, Clínica de Assistência à Mulher - Grupo CAM, Salvador, BA, Brazil.; 4 MD, PhD, Department of Medicine, Escola Bahiana de Medicina e Saúde Pública - Campus Brotas, Salvador, BA, Brazil.

**Keywords:** Magnetic resonance imaging, Breast neoplasms, Diffusion magnetic resonance imaging/methods, Neoplasms/pathology

## Abstract

**Objective:**

To test the use of diffusion-weighted imaging (DWI) in stratifying suspicious breast lesions (BI-RADS 4), correlating them with histopathology. We also investigated the performance of DWI related to the main enhancement patterns (mass and non-mass) and tested its reproducibility.

**Materials and Methods:**

Seventy-six patients presented 92 lesions during the sampling period. Two independent examiners reviewed magnetic resonance imaging studies, described the lesions, and determined the apparent diffusion coefficient (ADC) values. Differences among benign, indeterminate- to high-risk, and malignant findings, in terms of the ADCs, were assessed by analysis of variance. Using receiver operating characteristic (ROC) curves, we compared the performance of ADC values in masses and non-mass lesions, and tested the reproducibility of measurements by determining the coefficient of variation and smallest real difference.

**Results:**

Among the 92 lesions evaluated, the histopathology showed that 37 were benign, 11 were indeterminate- to high-risk, and 44 were malignant. The mean ADC differed significantly among those histopathological groups, the value obtained for the malignant lesions (1.10 × 10^-3^ mm^2^/s) being significantly lower than that obtained for the other groups (p < 0.001). ROC curves demonstrated that DWI performed better when applied to masses than when applied to non-mass lesions (area under the curve, 0.88 vs. 0.67). Reproducibility was good (coefficient of variation, 7.03%; and smallest real difference, ± 0.242 × 10^-3^ mm^2^/s).

**Conclusion:**

DWI can differentiate between malignant and nonmalignant (benign or indeterminate- to high-risk) lesions, showing better performance for masses. Nevertheless, stratification based on histopathological criteria that are more refined has yet to be achieved.

## Abstract (pt)

**Objetivo:**

Testar a sequência ponderada em difusão (SPD) para estratificação de lesões suspeitas (BI-RADS 4) por ressonância magnética em correlação com a histopatologia. Também investigamos o desempenho da SPD relacionada a padrões de realce (nódulo e não nódulo) e testamos sua reprodutibilidade.

**Materiais e Métodos:**

Setenta e seis pacientes apresentaram 92 lesões durante o período amostral. Dois examinadores independentes revisaram os estudos, descreveram as lesões e mediram os coeficientes de difusão aparente (CDAs). Diferenças de CDA entre achados benignos, indeterminados/de alto risco e malignos foram avaliadas por análise de variância. Comparamos o desempenho dos CDAs em nódulos e não nódulos por curvas *receiver operating characteristic* (ROC) e testamos a reprodutibilidade das mensurações pelo coeficiente de variação e menor diferença real.

**Resultados:**

Obtivemos 37 lesões benignas, 11 indeterminadas/de alto-risco e 44 cânceres. As médias dos CDAs desses grupos histopatológicos foram significativamente diferentes (p < 0,001), devido aos valores mais baixos em achados malignos (1,10 × 10^-3^ mm^2^/s). Curvas ROC demonstraram melhor desempenho da SPD aplicada a nódulos (área sob a curva de 0,88 contra 0,67 para não nódulos). A reprodutibilidade foi boa (coeficiente de variação de 7,03% e menor diferença real de ± 0,242 × 10^-3^ mm^2^/s).

**Conclusão:**

A SPD pode diferenciar achados malignos de não malignos, com melhor desempenho para nódulos. Entretanto, a estratificação baseada em critérios histopatológicos mais refinados ainda não foi alcançada.

## INTRODUCTION

Dynamic contrast-enhanced magnetic resonance imaging (DCE-MRI) has become established as the most sensitive method for breast cancer detection, with acceptable, albeit low, specificity^(1,2)^. New techniques applied to MRI, such as spectroscopy and diffusion-weighted imaging (DWI), have been producing encouraging results and have expanded the field for oncology studies^(3)^. Nevertheless, the use of these techniques has not yet achieved widespread clinical validation, because they are still considered ancillary tools in the evaluation of suspicious findings^(4)^.

Among the novel MRI procedures, DWI is regarded as one of the most promising methods of screening for malignancy and evaluating treatment response^(5)^. It has been shown that malignant neoplasms typically have lower apparent diffusion coefficients (ADCs) than do benign growths and normal features, in part due to the restricted extracellular space caused by the higher cell density in malignancies^(6)^. The measurement of ADCs might partially translate this microscopic complexity into a manageable quantitative parameter that can be used in order to distinguish among different biological tissues. In addition, most modern scanners are capable of employing DWI, which has short acquisition times, does not require the use of paramagnetic contrast medium, and, above all, has shown a potential to improve the specificity of MRI^(7)^.

The American College of Radiology Breast Imaging Reporting and Data System (BI-RADS) classifies suspicious abnormalities as category 4 (BI-RADS 4), with a wide variation in the risk of malignancy (> 2% to < 95%)^(8)^. As a consequence, BI-RADS 4 lesions require invasive investigation, which results in a varied spectrum of findings-from simple, nonproliferative changes to aggressive malignant tumors- leading to divergent clinical practices. In addition, histology reports of nonmalignant proliferative abnormalities with atypia and those with an indeterminate risk of malignancy usually prompt physicians to investigate more aggressively, which results in a high number of false-positive procedures^(9)^.

In its latest edition, the BI-RADS stratifies suspicious (category 4) lesions into three, narrower, subcategories (4A, 4B, and 4C), which respectively correspond to increasing positive predictive values for malignancy^(8)^. The approach is not infallible, given that positive predictive values vary according to prevalence^(10,11)^, and the subcategorization is currently applicable only to mammography and ultrasound. Because DWI provides information about the internal structure of living tissue^(6)^, it might improve the accuracy of DCEMRI by providing a better pathological correlation, which could ultimately engender a valid stratification of BI-RADS 4 lesions by histopathological subtype.

Our study aimed to determine whether ADC values could be used in order to establish a practical three-level histopathological classification of suspicious (BI-RADS 4) findings as benign, indeterminate- to high-risk, or malignant. We further probed the performance of DWI for mass and non-mass enhancement patterns, evaluating the reproducibility of measurements between examiners (interobserver reliability).

## MATERIALS AND METHODS

The subgroup of patients analyzed here was part of a larger cohort previously studied for different purposes^(12-14)^. The present study stems from a collaboration between an academic institution and a local private referral center for women's healthcare. An independent review board approved the study (Report no. 518466) and waived informed consent. Between November 2009 and December 2013, we performed 1973 breast MRI examinations at the private referral center. In 238 (12.1%) of those examinations, the lesion was classified as BI-RADS 4. All of the subjects were female and ≥ 18 years of age.

### Subjects and lesions

The study sample was defined according to the following inclusion criteria: DWI had been part of the imaging protocol; the histopathological correlation was available; the lesions were larger than 5.0 mm in diameter (foci were excluded); two reviewers had categorized the examination as technically adequate; and the DCE-MRI and DWI sequences had been properly restored. The examinations were anonymized with an inbuilt tool available in the Advantage Windows workstation, version 4.4 (GE Healthcare) and linked to a restricted version of the database managed by the private facility. Eighty examinations were incompletely restored or lost due to random computational storage problems (data corruption). Among the remaining 158 examinations, 199 lesions were identified. Pathology findings were unavailable for 89 lesions; 16 lesions were excluded due to field inhomogeneity and movement artifacts; and 2 lesions were excluded because they were considered too small. Therefore, the final study sample comprised a collective total of 92 lesions in 76 patients.

### MRI acquisition

All examinations were performed with patients in the prone position in a single 1.5-T MRI scanner (Signa Excite HDxT; GE Healthcare, Waukesha, WI, USA), with an 8-channel phased-array bilateral breast coil. In our protocol, the DWI sequence comes last, immediately after the contrast-enhanced sequences, and consists of single-shot echo-planar acquisition in the axial plane (repetition time/echo time, 11.7/96; number of excitations, 8; matrix size, 256 × 224; field of view, 340 × 340 mm; slice thickness, 3.5 mm; intersection gap, 0.5 mm), together with array spatial sensitivity encoding technique parallel imaging. Diffusion gradients are applied in six directions with b = 0 and 750 s/mm^2^. The remaining sequences-T1-weighted fast spin-echo; T2-weighted fat-suppressed fast spin-echo; and post-contrast dynamic T1-weighted fat-suppressed images using Volume Image Breast Assessment (VIBRANT) technique (GE Health-care), after an intravenous bolus injection of gadoterate meglumine (Dotarem, 0.1 mmol/kg of bodyweight; Guerbet, Roissy, France)-are all acquired in the sagittal plane. If the patient is compliant, we also obtain a single-phase late axial isotropic fat-suppressed T1-weighted sequence using VIBRANT. The detailed parameters of our protocol can be accessed in previous publications^(12,14)^.

### Image assessment

The restored examinations were anonymized and kept in an offline Advantage Windows workstation, version 4.4 (GE Healthcare), with the FuncTool software package (GE Healthcare) and full post-processing capability. Two radiologists with at least five years of experience and 1000 breast MRI readings to their credit, who were blinded to all clinical and pathological data, independently reassessed the images, with subsequent consensual analysis of discordant cases. They were instructed to evaluate the standard acquisitions (non-contrast-enhanced and DCE-MRI sequences) and to correlate the suspicious findings with the corresponding areas of high signal intensity on DWI sequences. Gray-scale ADC maps were generated based on the following equation:

ADC=−l/bln(S/DWIS)0

where *b* = 750 s/mm^2^; *S_DWI_* is the geometric mean of the individual *b* = 750 s/mm^2^ images obtained with DWI; and *S_0_* is the *b* = 0 s/mm^2^. At least two circular or oval regions of interest (ROIs) were manually placed on the suspicious areas, including a minimum of four pixels, with independent averages calculated to test interobserver variability.

### Pathological evaluation

In the restricted version of our electronic database, histopathological reports, most of them produced at our facility, were available for all of the examinations evaluated. We consulted with an experienced pathologist subspecializing in breast diseases and devised a three-level histological classification with practical clinical implications^(15)^. The pathologist reviewed the reports and stratified the lesions in the following manner: benign (nonproliferative and proliferative lesions without atypia); indeterminate- to high-risk (proliferative lesions with atypia and those of unknown malignant potential, pending a larger tissue sample, which comprise papillary and complex sclerosing lesions); and malignant (any type of invasive carcinoma or *in situ* ductal carcinoma). In samples with mixed pathological features, the most clinically relevant finding would dictate the stratification (e.g., fibrocystic changes with concurrent atypical hyperplasia would be included in the indeterminate- to high-risk group and lobular neoplasia mixed with invasive lobular carcinoma would be included in the malignant group).

Biological specimens were obtained by surgical excision, core biopsies being performed with a spring-loaded reusable core biopsy system device (Bard Magnum; Bard Biopsy Systems, Tempe, AZ, USA), using 14-gauge needles, and vacuum-assisted biopsies being performed with a 9-gauge probe (ATEC; Suros Surgical Systems, Indianapolis, IN, USA). All diagnoses of indeterminate- to high-risk lesions were eventually confirmed by surgery. For practical and financial reasons, when there was good imaging correlation with MRI, preoperative localization and biopsy procedures were preferably guided by mammography or ultrasound. Therefore, direct MRI guidance was used in only three preoperative localizations and one vacuum-assisted biopsy.

All of the participants with biopsy-negative lesions that were not explored further by surgery were followed clinically and through imaging studies for at least two years. When there was radiological-pathological discordance after the biopsy, surgical excision was performed, and the result of the investigation was categorized accordingly.

### Statistical analysis

We collected data on age, lesion size, main enhancement pattern (mass or non-mass), and ADC values (for the breast parenchyma and lesions), as well as histopathological results, which were categorized in three groups (benign, indeterminate- to high-risk, and malignant). Data are reported as absolute and relative frequencies, measures of central tendency (means and medians), and measures of dispersion-standard deviations (SDs) and interquartile ranges (IQRs)-when appropriate.

Data related to participant ages and lesion sizes were checked for normality, and, because the null hypothesis was rejected, we used the Kruskal-Wallis test with Dunn's pairwise *post hoc* comparisons. We also used the Shapiro-Wilk test to determine the distribution of ADC values (*p* = 0.17), and Levene's robust test to determine groupwise homoscedasticity (*p* = 0.33), subsequently applying analysis of variance with Scheffé correction. The ADC values for the breast parenchyma also did not deviate significantly from the standard normal distribution (*p* = 0.10) and were compared with those of lesions by paired t-test. We then used analysis of variance to assess the differences in ADCs between mass and non-mass enhancement patterns among the histopathological groups. Receiver operating characteristic (ROC) curves were generated to compare the performance of DWI in using the enhancement pattern to differentiate malignant lesions from indeterminate- to high-risk or benign (hereafter collectively referred to as nonmalignant) lesions. The area under the curve (AUC), with a 95% confidence interval (95% CI), was calculated for each pattern. We also applied Pearson's chi-square test to determine whether mass and non-mass enhancement patterns were independently linked to any particular histopathological group. The reproducibility of ADC values was determined by calculating the coefficient of variation between the examiners^(16)^. We calculated the smallest real difference, which is similar to the Bland-Altman limits of agreement^(17)^ and provides a measure of the relevant change^(18)^.

Considering a total sample size of 92, a calculated effect size of 0.39, and a variance roughly equal to 0.37, we estimated the *post hoc* power of the study for its primary objective to be greater than 90%. We acknowledge that, for a small number of patients, there was more than one result (1.21 lesions per subject). Nevertheless, those few correlated outcomes are not expected to have significant impact on our results, as previously indicated^(19)^ and as suggested by a cursory sensitivity analysis with robust standard errors. All computations were performed with Stata statistical software, version 12.0 (StataCorp LP; College Station, TX, USA), except for the power calculations, for which we used G*Power, version 3.1.9.2 (Faul, Erdfelder, Lang and Buchner, 2006, 2009). Values of *p* < 0.05 were considered statistically significant for two-tailed tests.

## RESULTS

Of the 92 lesions evaluated, 37 (40.2%) were categorized as benign, 11 (12.0%) were categorized as indeterminate- to high-risk, and 44 (47.8%) were categorized as malignant. The histopathological results were mostly related to material obtained from surgical excisions, corresponding to 68 (73.9%) of the specimens, whereas the remaining material was obtained from core-needle biopsies and vacuum-assisted biopsies, which accounted for 20 (21.7%) and 4 (4.4%) of the specimens, respectively. There were no significant differences among the various tissue acquisition techniques in terms of the pathological results (*p* = 0.456). The overall median age of the patients was 51 years (IQR, 42-59 years), and the median lesion size was 1.6 cm (IQR, 1.0-3.7 cm). As can be seen in Table 1, there were no significant differences among groups in terms of patient age (*p* = 0.229), although the groups differed significantly in terms of lesion size, which was greatest in the malignant group (*p* = 0.006). The median lesion size was comparable between the benign and indeterminate- to high-risk groups, which could not be distinguished by pairwise comparison (*p* = 0.182).

**Table 1:** Characteristics of the patients and lesion enhancement patterns by histological group.

Characteristic	Benign	Indeterminate-to high-risk	Malignant	*p* *
Proportion of total	40.2%	12.0%	47.8%	—
Age, in years †	47 (32-55)	53 (40-73)	54 (43-62)	0.229
Lesion size, in cm †	1.3 (0.9-2.5)	0.9 (0.8-1.5)	2.2 (1.5-4.7)	0.006
Enhancement pattern ‡				0.967
Mass	17 (41.5)	5 (12.2)	19 (46.3)	—
Non-mass	20 (39.2)	6 (11.8)	25 (49.0)	—

* Kruskal-Wallis test for age and lesion size; Pearson's chi-square test for enhancement patterns.

† Median (interquartile range).

‡ Number of lesions (proportion of enhancement pattern).

**Figure 1:**
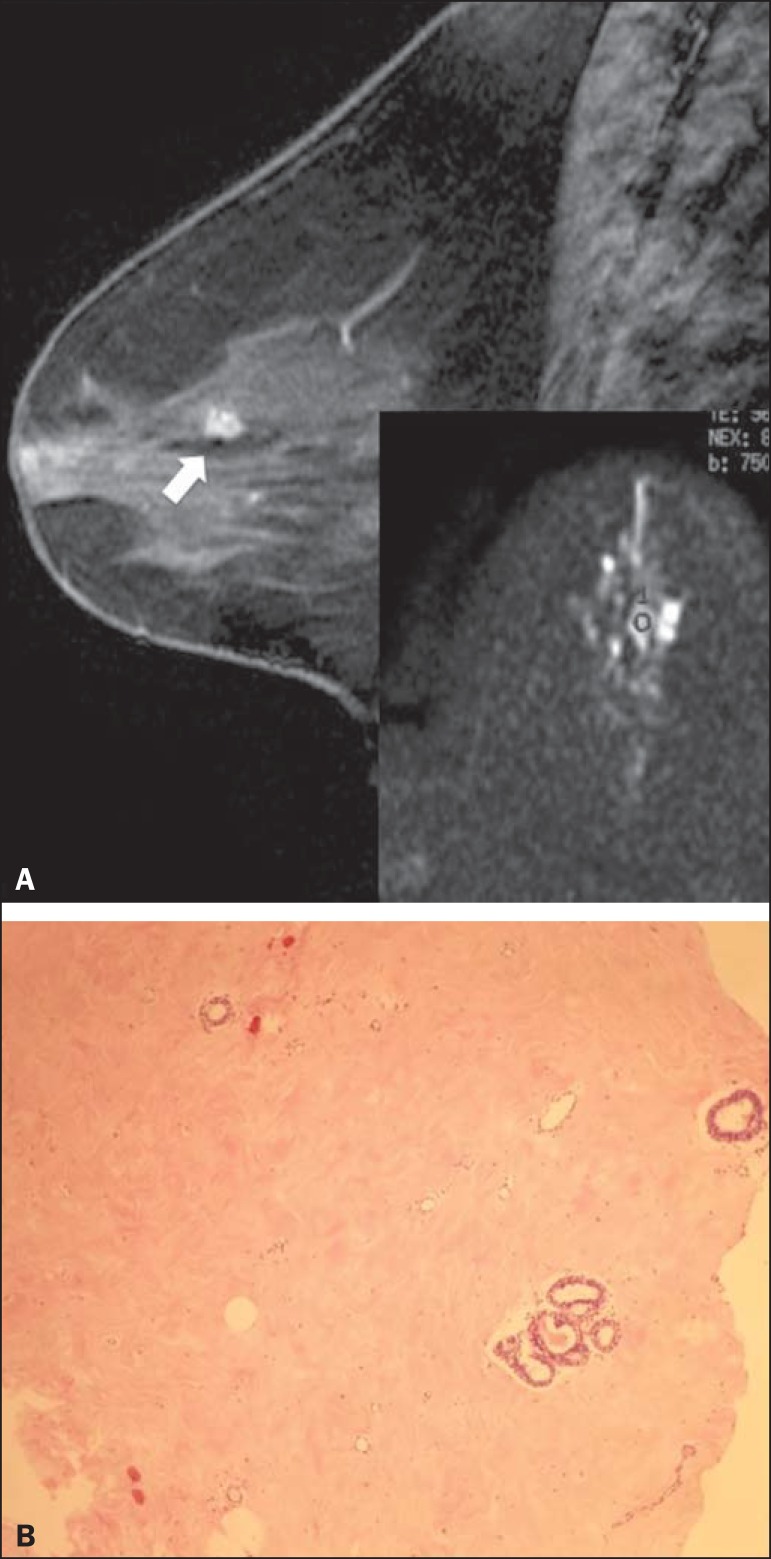
Contrast-enhanced sagittal T1-weighted fat-saturated MRI scan (**A**) showing a 0.8-cm focal enhancement considered suspicious (arrow) and its representation on axial DWI (marked with an ROI), which displays an ADC of 1.30 × 10^-3^ mm^2^/s. Photomicrograph (**B**) of a specimen obtained by vacuum-assisted biopsy showing dense fibrous stroma on greater magnification (hematoxylin-eosin staining).

**Figure 2:**
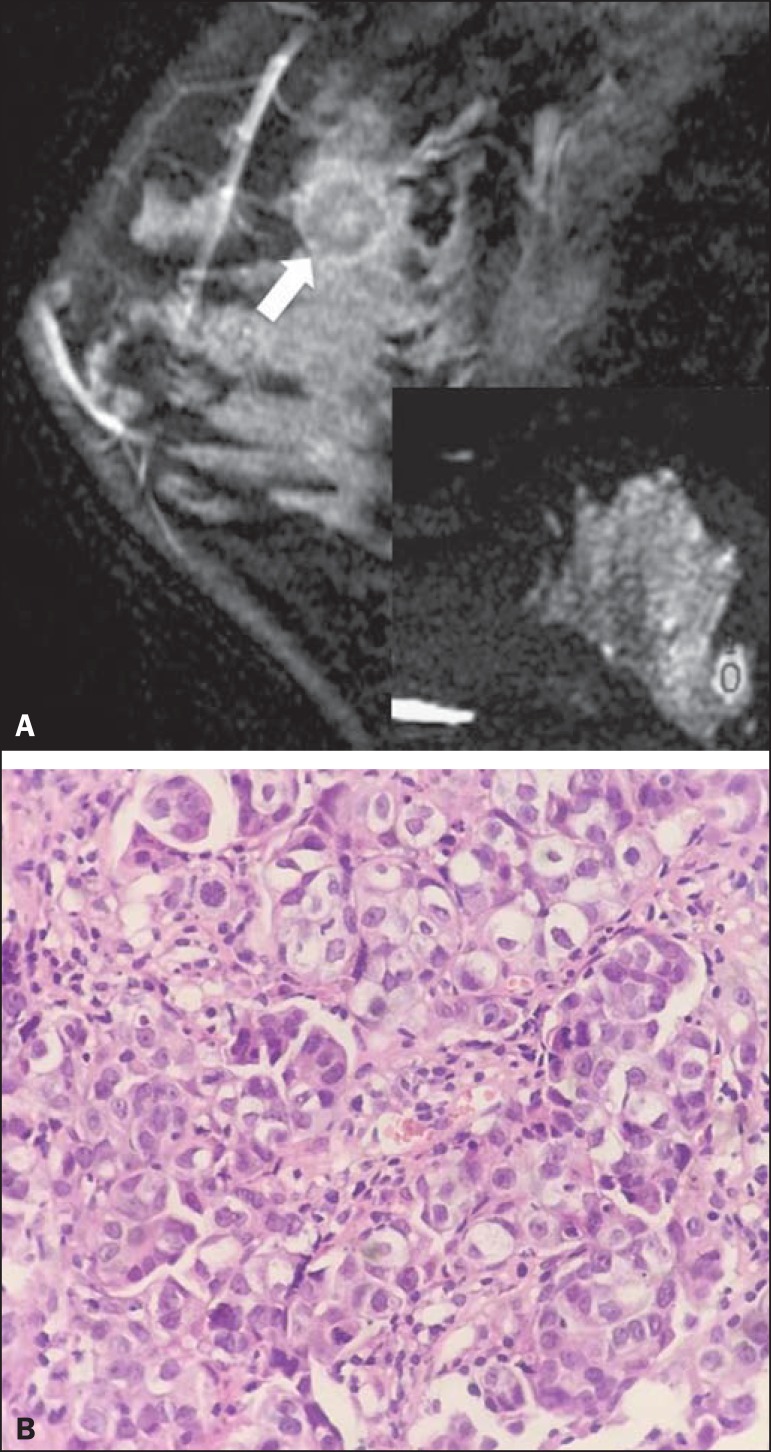
Contrast-enhanced sagittal T1-weighted fat-saturated MRI scan (**A**) showing a 1.6-cm round mass (arrow), with irregular margins, and the corresponding DWI (marked with an ROI), with an ADC of 0.74 × 10^-3^ mm^2^/s. Photomicrograph (**B**) of a specimen obtained by ultrasound-guided core biopsy showing clusters of malignant, high nuclear grade (grade 3) epithelial cells and lymphocytic infiltrate on greater magnification (hematoxylin-eosin staining).

The overall ADC value (mean ± SD) for the breast parenchyma and lesions was 1.83 ± 0.30 × 10^-3^ mm^2^/s and 1.25 ± 0.034 × 10^-3^ mm^2^/s, respectively (*p* < 0.001). There were also significant differences among the groups in terms of the mean ADC, which was lowest (1.10 ± 0.309 × 10^-3^ mm^2^/s) in the malignant group (*p* < 0.001), although it was similar between the benign and indeterminate- to high-risk groups (pairwise comparison, *p* = 0.972). Table 2 provides detailed descriptive data on ADC values by pathological group and specific diagnosis. Figures 1 and 2 show examples of lesions considered suspicious on the basis of their imaging characteristics (BI-RADS 4 lesions) but with distinct histopathology.

**Table 2:** ADC values by histological group and specific pathological result, in relation to the 1.21 x 10 3 mm2/s cut-off point, among the 92 lesions evaluated.

			ADC				
			< 1.21 x 10^-3^ mm^2^/s			≥ 1.21 x 10^-3^ mm^2^/s	
Group	Number of lesions	Mean ± SD	N	%*		N	%*
Benign	37	1.38 ± 0.25	11	29.7		26	70.3
Adenosis	4	1.35 ± 0.19	1	25.0		3	75.0
Fibrocystic changes	4	1.41 ± 0.31	2	50.0		2	50.0
Ductal ectasia	2	1.68 ± 0.32	0	0		2	100.0
Fibroadenoma	12	1.47 ± 0.27	2	17.0		10	83.0
Fibrosis	5	1.28 ± 0.07	0	0		5	100.0
Hypervascularity†	1	1.15	1	100.0		0	0
Usual ductal hyperplasia	4	1.25 ± 0.15	2	50.0		2	50.0
Inflammation	3	1.12 ± 0.07	3	100.0		0	0
Pseudoangiomatous stromal hyperplasia	2	1.58 ± 0.30	0	0		2	100.0
Indeterminate- to high-risk	11	1.41 ± 0.37	3	27.3		8	72.7
Atypical hyperplasia	1	1.29	0	0		1	100.0
Complex sclerosing lesion	1	1.05	1	100.0		0	0
Papillary lesion	9	1.46 ± 0.38	2	22.0		7	78.0
Malignant	44	1.10 ± 0.31	31	70.5		13	29.6
Invasive ductal carcinoma	22	0.96 ± 0.21	21	95.5		1	4.5
Invasive lobular carcinoma	6	1.15 ± 0.17	3	50.0		3	50.0
Neuroendocrine carcinoma	2	0.96 ± 0.63	1	50.0		1	50.0
Invasive mucinous carcinoma	2	1.83 ± 0.53	0	0		2	100.0
*In situ* ductal carcinoma	12	1.23 ± 0.23	6	50.0		6	50.0

* Percentages may not total 100, because of rounding.

† Description provided by the pathologist after correlation with the MRI findings.

**Figure 3:**
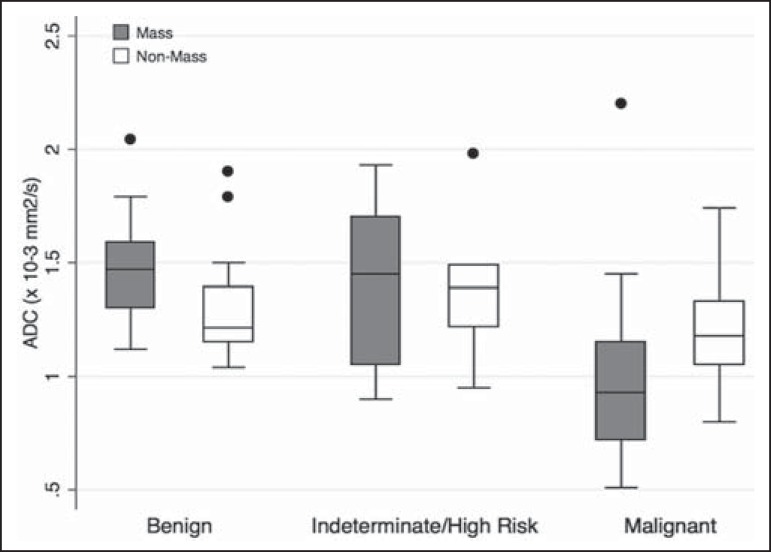
ADC values of masses (dark gray boxes) and non-mass enhancement (white boxes), stratified by histological group, black circles indicating extreme values. Significantly lower median ADCs (central lines in box plots) are observed for malignant masses (*p* < 0.001). Lesions with non-mass enhancement could not be confidently differentiated by their ADC values (*p* = 0.071).

**Figure 4:**
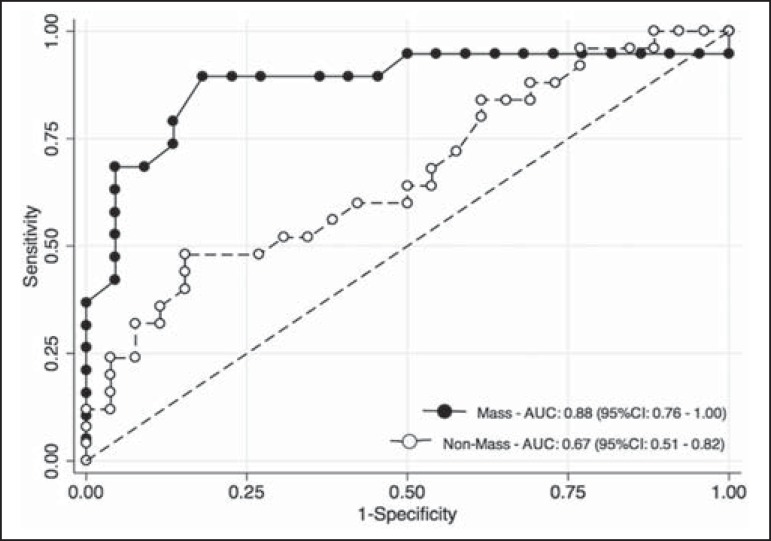
ROC curves for mass and non-mass enhancement patterns. The AUC, which corresponds to the probability of correctly classifying a lesion as malignant or nonmalignant (benign or indeterminate- to high-risk), indicated that ADC values perform better when applied to masses (AUC, 0.88) than when applied to nonmass enhancement (AUC, 0.67), the difference being statistically significant (*p* = 0.029).

Among the histopathological groups, the two main enhancement patterns were proportionally distributed and showed discrepant ADC values (Figure 3). Only the lesions with mass enhancement could be reliably stratified (*p* < 0.001), the pairwise comparisons showing that the malignant group differed from the benign and indeterminate- to high-risk groups (*p* = 0.001 and *p* = 0.059, respectively). On the basis of the ADC values, it was not possible to separate the non-mass enhancement patterns into malignant and nonmalignant types (*p* = 0.071), although the AUCs indicated that the ADC performed significantly better in that regard for the mass enhancement patterns (Figure 4).

On the basis of data previously published^(12)^, we employed an ADC cut-off point of 1.21 × 10^-3^ mm^2^/s, values lower than that being considered diagnostic of malignancy. Thus, 31 (70.5%) of the 44 lesions in the malignant group were classified as malignancies, whereas 26 (70.3%) of the 37 lesions in the benign group showed an ADC above the cut-off point, as did 8 (72.7%) of those in the indeterminate-to high-risk group (Table 2).

The interobserver variability for ADC measurements was considered small, with a mean difference between examiners of ± 0.123 × 10^-3^ mm^2^/s (SD of ± 0.019 × 10^-3^ mm^2^/s) and an overall coefficient of variation of 7.03%. To be considered relevant, a discrepancy between any two measurements had to be outside the limits of ± 0.242 × 10^-3^ mm^2^/s, according to the smallest real difference calculated. The examiners produced only three such pairs of measurements.

## DISCUSSION

Our results support the notion that DWI can be used in order to differentiate between malignant tumors and nonmalignant abnormalities. However, benign and indeterminate- to high-risk subtypes were indistinguishable in our sample, precluding a more detailed classification based on histopathological features. The interobserver variability for ADC values was small, given that only three pairs of measurements were considered substantially discordant.

We found that the mean ADC was lower for any type of lesion than for the normal breast parenchyma. In the present study, the mean ADC was lowest in the malignant group, a finding supported by those of previous studies^(20,21)^. A review article authored by Tsushima et al.^(22)^ showed that DWI performs well in the diagnosis of breast cancer, with a pooled sensitivity and specificity of 0.89 and 0.77, respectively, similar to that shown by Qu et al.^(23)^ in a more recent meta-analysis. In the present study, DWI performed similarly-with an estimated accuracy (AUC) of 0.88-although only for mass enhancement patterns. However, the AUC estimated for non-mass enhancement patterns was less than optimal (0.67), comparable to the 0.70 reported for such patterns by Imamura et al.^(24)^ and virtually identical to the 0.66 estimated by Partridge et al.^(25)^. One plausible explanation for the discrepancy between the mass and non-mass enhancement patterns is that, in the latter pattern, normal breast parenchyma is typically intermingled with pathological tissue, inevitably leading to averaged ADC measurements, even if multiple ROIs are evaluated.

There have been few studies concerning DWI analysis of more detailed histological subgroups^(26,27)^. Parsian et al.^(27)^ distinguished high-risk findings from benign breast lesions by their mean ADCs after a false-positive MRI classification (BIRADS 4 or 5). Nevertheless, the authors did not find a statistically significant difference between high-risk and malignant lesions^(27,28)^. This is in contradistinction to our findings, because we observed a substantial overlap between the nonmalignant subtypes (benign and indeterminate- to high-risk lesions) in term of the ADCs, which were clearly distinct from those of malignant lesions. That might be explained by differences in the histopathological classification. In order to achieve a practical, clinically meaningful categorization, we grouped high-risk atypical lesions together with those of indeterminate malignant potential, because both usually elicit further investigation. In addition, we identified a small number of proliferative changes with atypia and no lobular neoplasia (atypical lobular hyperplasia or lobular carcinoma in situ). Nevertheless, in our indeterminate- to high-risk group, the mean ADC was quite close to that reported by Parsian et al.^(27)^-a difference of only 0.05 × 10^-3^ mm^2^/s-whereas, in our benign and malignant groups, it was substantially lower than the values reported by those authors.

A number of ADC cut-off points have been tested, for a variety of purposes^(20,27,28)^. In a study previously conducted by our group^(12)^, we used a balanced cut-off point of 1.21 × 10^-3^ mm^2^/s to separate malignant from nonmalignant findings, thus correctly classifying approximately 70% of the lesions. We acknowledge that adopting a higher ADC cut-off point would be a better strategy to safely avoid more aggressive investigation of BI-RADS 4 findings. In our sample, all lesions with ADC values above 1.74 × 10^-3^ mm^2^/s were determined to be nonmalignant, except for one mucinous carcinoma (ADC, 2.20 × 10^-3^ mm^2^/s), which corresponded to 7.6% of the findings. This cut-off point is close to the arithmetic mean of the values reported by other authors, which have ranged from 1.60 to 1.81 × 10^-3^ mm^2^/s. Spick et al.^(29)^ also stated that DWI might obviate the need for MRI-guided biopsies. In their study, 34.5% of the false-positive procedures would have been avoided, without false-negatives, if a cut-off point of 1.58 × 10^-3^ mm^2^/s had been adopted.

The reproducibility of measurements in the present study was considered good, because the mean difference was small and the coefficient of variation was below 10%. The discordance was considered clinically relevant, based on the smallest real difference, in only three cases. This finding is supportive of those of other authors, who have reported high or very high interobserver reliability of ADC measurements when trained examiners were involved^(30)^.

To our knowledge, this is the first study aiming to stratify suspicious-only (BI-RADS 4) lesions by DWI according to detailed histopathological features, thus avoiding the simple benign vs. malignant dichotomy. By doing so, we were able to devise different recommendations according to the primary biopsy results: biopsy-proven benign lesions could be followed by imaging methods; indeterminate- to high-risk findings would require additional clinical and pathological correlation, because they might be upgraded after analysis of tissue samples that are more representative; and malignancies could be treated without delay.

This study has some limitations. Our objective was to stratify BI-RADS 4 lesions according to tissue characteristics, with comprehensive clinical implications. Therefore, to simulate the three subcategories already established for other imaging methods (4A, 4B, and 4C), we grouped findings with indeterminate malignant potential together with those categorized as high-risk. This strategy led to different histological subtypes (ranging from typically benign to atypical) being grouped together while a more definitive pathological evaluation was pending. As a consequence, the variability of ADC measurements in that group could have been increased. In addition, in our sample, there were relatively few high-risk findings and no lobular neoplasias. We also understand that, because this was a single-center study, with examiners routinely discussing cases seen in their daily clinical practice, the high level of interobserver agreement was to be expected.

In summary, we have demonstrated that DWI performs well in differentiating malignant lesions from nonmalignant abnormalities even in the challenging subgroup of patients with suspicious (BI-RADS 4) lesions. A more detailed stratification based on ADC values of representative histopathological characteristics might be feasible, although studies involving larger samples would be needed.
